# Transcriptome Analysis Reveals Catabolite Control Protein A Regulatory Mechanisms Underlying Glucose-Excess or -Limited Conditions in a Ruminal Bacterium, *Streptococcus bovis*

**DOI:** 10.3389/fmicb.2021.767769

**Published:** 2021-11-18

**Authors:** Yaqian Jin, Yaotian Fan, Hua Sun, Ying Zhang, Hongrong Wang

**Affiliations:** Laboratory of Metabolic Manipulation of Herbivorous Animal Nutrition, College of Animal Science and Technology, Yangzhou University, Yangzhou, China

**Keywords:** *Streptococcus bovis* S1, catabolite control protein A, transcriptome, glucose concentration, metabolism regulation

## Abstract

Ruminants may suffer from rumen acidosis when fed with high-concentrate diets due to the higher proliferation and overproduction of lactate by *Streptococcus bovis*. The catabolite control protein A (CcpA) regulates the transcription of lactate dehydrogenase (*ldh*) and pyruvate formate-lyase (*pfl*) in *S. bovis*, but its role in response to different carbon concentrations remains unclear. To characterize the regulatory mechanisms of CcpA in *S. bovis* S1 at different levels of carbon, herein, we analyzed the transcriptomic and physiological characteristics of *S. bovis* S1 and its *ccpA* mutant strain grown in glucose-excess and glucose-limited conditions. A reduced growth rate and a shift in fermentation pattern from homofermentation to heterofermentation were observed under glucose-limited condition as compared to glucose-excess condition, in *S. bovis* S1. Additionally, the inactivation of *ccpA* significantly affected the growth and end metabolites in both conditions. For the glycolytic intermediate, fructose 1,6-bisphosphate (FBP), the concentration significantly reduced at lower glucose conditions; its concentration decreased significantly in the *ccpA* mutant strain. Transcriptomic results showed that about 46% of the total genes were differentially transcribed between the wild-type strain and *ccpA* mutant strain grown in glucose-excess conditions; while only 12% genes were differentially transcribed in glucose-limited conditions. Different glucose concentrations led to the differential expression of 38% genes in the wild-type strain, while only half of these were differentially expressed in the *ccpA*-knockout strain. Kyoto Encyclopedia of Genes and Genomes (KEGG) enrichment analyses showed that the substrate glucose concentration significantly affected the gene expression in histidine metabolism, nitrogen metabolism, and some carbohydrate metabolism pathways. The deletion of *ccpA* affected several genes involved in carbohydrate metabolism, such as glycolysis, pyruvate metabolism, fructose and mannose metabolism, as well as in fatty acid biosynthesis pathways in bacteria grown in glucose-excess conditions; this effect was attenuated under glucose-limited conditions. Overall, these findings provide new information on gene transcription and metabolic mechanisms associated with substrate glucose concentration and validate the important role of CcpA in the regulation of carbon metabolism in *S. bovis* S1 at differential glucose availability.

## Introduction

In ruminants, undesirable lactate accumulation in the rumen due to high-concentrate diets can cause rumen acidosis. Previous studies show that *Streptococcus bovis*, in presence of sufficient highly digestible carbohydrates, proliferates rapidly, and predominates over a short period in the rumen with the accumulation of lactate as the major fermentation product (Marounek and Bartos, 1987; Nocek, 1997). This causes a large drop in ruminal pH (Kenney et al., 2015). Thus, *S. bovis* may play an important role in the progress of rumen acidosis. A better understanding of the factors affecting lactate production and overgrowth of *S. bovis* will be beneficial for preventing rumen acidosis.

*Streptococcus bovis*, an amylolytic and lactate-producing bacterium in the rumen, can rapidly break down starch into glucose and produce lactate, formate, acetate, and ethanol as the end metabolites (McAllister et al., 1990; Asanuma and Hino, 2002b). Following the Embden–Meyerhof–Parnas (EMP) pathway, pyruvate in *S. bovis* is either metabolized to lactate, by lactate dehydrogenase (LDH) or to formate and acetyl-CoA by pyruvate formate-lyase (PFL) (Russell and Hino, 1985); acetyl-CoA is converted subsequently to acetate or ethanol. Thus, the proportion of organic acids produced by *S. bovis* is dependent on the activity ratio of LDH to PFL (Asanuma et al., 1999; Asanuma and Hino, 2000). The activities of LDH and PFL are not only dependent on some glycolytic intermediates, such as fructose 1,6-bisphosphate (FBP), glyceraldehyde 3-phosphate (GAP), and dihydroxyacetone phosphate (DHAP) (Asanuma and Hino, 1997, 2002a), but also on the amount of enzyme protein, which are affected by energy supply and the intracellular pH at the transcription level (Asanuma et al., 1997, 1999; Asanuma and Hino, 2000). When the pH is low and the glucose is sufficiently available, the specific activity and amount of LDH increase, while the PFL synthesis and activity decrease (Asanuma et al., 1999), causing higher lactate production and lesser production of acetate, ethanol, and formate.

Additionally, the synthesis of LDH and PFL in *S. bovis* is also controlled at the transcriptional level by the catabolite control protein A (CcpA), which give a potential to control rumen acidosis due to overproduction of lactate by *S. bovis* at high-concentrate diets (Asanuma et al., 2004a). CcpA is a pleiotropic regulatory protein in low-GC Gram-positive bacteria and has a key role in the regulation of carbon and nitrogen metabolism, biofilm formation, toxic gene expression, and other physiological processes (Li et al., 2016). In *S. bovis* 12U1, the *ldh* mRNA level and LDH specific activity of the *ccpA*-knockout strain were significantly lower than those of the wild-type strain when the bacteria were grown on glucose; however, there were no significant difference when lactose was used as the substrate (Asanuma et al., 2004a). These findings show that the target gene regulation by CcpA depends on the source of carbon. However, whether the regulation of CcpA on the production of organic acids and expression of target genes is dependent on energy availability, remains unknown.

In the present study, to better understand the global regulation of CcpA in the carbohydrate metabolism of *S. bovis* S1 in response to carbon availability, the transcriptomic and physiological characteristics were analyzed for *S. bovis* S1 and its *ccpA* mutant strain grown under glucose-excess or glucose-limited conditions. Based on these results, we identified a large number of genes controlled by CcpA at different carbon substrate concentrations, thereby revealing further details of CcpA-mediated regulatory networks in *S. bovis* S1.

## Materials and Methods

### Bacterial Strains and Growth Conditions

*Streptococcus bovis* S1 used in this study was previously isolated from the rumen fluid of Saanen goats in our laboratory (Chen et al., 2016b). The *ccpA* mutant of *S. bovis* S1 was constructed by homologous recombination in this experiment. Before inoculation, both the *S. bovis* S1 and its *ccpA* mutant were revived in a modified de Man, Rogosa and Sharpe (MRS) medium (Chen et al., 2016b) in an anaerobic workstation (DG250, Don Whitley Scientific, England) at 37°C. These cultures (at exponential phase) were transferred using 1% (v/v) inoculum into 200 mL anaerobic serum bottles containing 100 mL basal medium, respectively. The basal medium was prepared according to the methods described in Chen et al. (2016b); and it contained: 0.45 g/L KH_2_PO_4_, 0.9 g/L NaCl, 0.9 g/L (NH_4_)_2_SO_4_, 0.12 g/L CaCl_2_⋅2H_2_O, 0.19 g/L MgSO_4_⋅7H_2_O, 1.0 g/L tryptone, 1.0 g/L yeast extract, and 0.6 g/L cysteine hydrochloride. Glucose solutions were filter-sterilized and added to the sterile basal medium at a final concentration of 5 or 50 mM. After inoculation, the culture bottles were sealed and transferred to a thermostat shaker (TS-1102C, Bosheng Scientific Instrument Co., Ltd., Yangzhou, China) and grown at 37°C and 160 rpm. The pH of the medium was constantly maintained at 6.5 by continuous titration with 10% NaOH. Three replicates were set for each treatment.

### Construction of *ccpA* Mutant

The *ccpA* gene was disrupted by homologous recombination as follows. First, DNA fragments corresponding to the upstream (1053 bp fragment; primer pairs *ccpA* Up F/*ccpA* Up R, Supplementary Table 2) and downstream (1101 bp fragment; primer pairs *ccpA* Down F/*ccpA* Down R, Supplementary Table 2) sequences of *ccpA* were amplified by PCR using *S. bovis* S1 genomic DNA as a template. The erythromycin resistance gene *erm* was amplified with the primers *erm* F and *erm* R (Supplementary Table 2). The PCR product was purified using a PCR purification kit (Qiagen, Beijing, China) according to the manufacturer’s instructions. The amplified fragments were, respectively, cloned into *Eco*RI, *Bam*HI, and *Sac*I restriction sites of pUC19 vector to generate pUC19-*ccpA* up-*erm*-*ccpA* down (pCE). The recombinant vector pCE was electroporated into *S. bovis* S1 cells using an electroporation system at 2.5 kV, 200 Ω, and 25 μF. Finally, knockout mutants were selected on MRS plates containing 1 μg/mL erythromycin at 37°C for 3–4 days. The results of knockout were validated by qRT-PCR and DNA sequencing as previously described (Chen et al., 2015; Supplementary Figure 1).

### Sample Collection

Cell growth was monitored by measuring OD values at 600 nm using SpectraMax M5 plate reader (Molecular Devices Corporation, United States) at 1-h intervals, and the maximal growth rate (μ_max_) was estimated according to the logistic model (Perni et al., 2005; Dai et al., 2020). The cultures of each sample were harvested by centrifugation (12,000 rpm, 2 min, 4°C) when they reached exponential phase (at OD_600_ of 0.6 for the wild-type and *ccpA* mutant strains grown in glucose-excess condition, at OD_600_ of 0.2 for both strains grown in glucose-limited condition) and stationary phase (at OD_600_ of 0.9 for both strains grown in glucose-excess condition, at OD_600_ of 0.3 for both strains grown in glucose-limited condition). The cell pellets obtained at exponential phase were quickly frozen in liquid nitrogen for 15 min, and stored at −80°C for further RNA isolation; the supernatants at both growth phases were filtered using a 0.22 μm filter membrane and stored at −80°C for the determination of metabolites.

### Analysis of Metabolites

A high-performance liquid chromatographer (HPLC, Shimadzu, Japan) equipped with an acclaim OA column (Sepax Carbomix H-NP) and a UV detector was used to detect concentrations of organic acids (lactate, formate, and acetate) in the supernatant. The column temperature was maintained at 55°C; the mobile phase was 2.5 mM H_2_SO_4_, and its flow rate was set at 0.5 mL/min. Organic acids were then measured with a UV detector set at 210 nm. The concentration of FBP was determined using a commercial kit (Comin Biotechnology Co., Ltd., Suzhou, China), according to the manufacturer’s instructions.

### RNA Extraction and Transcriptomic Analysis

Total RNA from *S. bovis* S1 wild-type and mutant strains, grown in the different conditions were extracted using TRIzol reagent (Invitrogen, Shanghai, China) according to the manufacturer’s instructions. The quality and integrity of total RNA were determined using NanoDrop spectrophotometer (Thermo Scientific, United States) and Bioanalyzer 2100 system (Agilent Technologies, Palo Alto, CA, United States). Total RNA was purified using Zymo-Seq RiboFree Total RNA Library Kit. The first-strand cDNA was synthesized using random oligonucleotides and SuperScript III and the second-strand cDNA synthesis was subsequently performed using DNA Polymerase I and RNase H. Remaining overhangs were converted into blunt ends by exonuclease/polymerase activities and the enzymes were removed. After adenylation of the 3′ ends of the DNA fragments, Illumina PE adapter oligonucleotides were ligated for subsequent hybridization. To select cDNA fragments of the preferred length (400–500 bp), the library fragments were purified using the AMPure XP system (Beckman Coulter, Beverly, CA, United States). DNA fragments with ligated adaptor molecules on both ends were selectively enriched using Illumina PCR Primer Cocktail in a 15-cycle PCR reaction. Products were purified (AMPure XP system) and quantified using the Agilent high sensitivity DNA assay on a Bioanalyzer 2100 system (Agilent Technologies, Palo Alto, CA, United States). The library obtained was sequenced on NovaSeq 6000 platform (Illumina) by Shanghai Personal Biotechnology Co., Ltd.

### RNA-seq Analysis

The raw data was filtered using Cutadapt (v1.15) software to obtain high-quality data, free of adapter sequences, primers, poly-A tails and unwanted artifacts from the high-throughput sequencing reads. The filtered reads were mapped onto the reference genome of *Streptococcus equinus* S1, using Bowtie2.^1^ The gene read count value was calculated using HTSeq (v0.9.1) as the original expression level of the gene. To normalize the gene expression levels of different genes and different samples, fragments per kilobase of exon per million fragments mapped (FPKM) was used. DESeq (v1.30.0) was used to analyze the differentially expressed mRNA transcripts with | log_2_FoldChange | > 1. *P*-value < 0.05 was considered as a statistically significant differential expression.

### Validation of Transcriptomic Results Using qPCR

Primers for qRT-PCR were designed using Beacon Designer 7.0 software. Total RNA was isolated using the method described above, and reverse-transcribed to cDNA using Quant reverse transcriptase (Tiangen, Biotech Co., Ltd., China), as per the manufacturer’s instructions. The qPCR was performed on the ABI Step-One-Plus RT-PCR system (ABI 7500, Applied Biosystems, Foster City, CA) using the TB Green Premix Ex Taq™ II Kit (TaKaRa Biotechnology Co., Ltd., Dalian, China). A 20 μL reaction was set; reaction solution contained 10 μL 2 × TB Green Premix Ex Taq II, 1.6 μL primer, 1 μL cDNA, 0.4 μL 50 × ROX, and 7.0 μL Rnase-free water. The RT-qPCR conditions were as follows: 95°C for 30 s, followed by 40 cycles of the amplification at 95°C for 5 s and 60°C for 34 s. All samples were evaluated in triplicates. Relative gene expression was normalized with the expression of the *16S* rRNA gene and calculated using the 2^–ΔΔCT^ method.

### Statistical Analyses

Results were expressed as “mean ± SD (standard deviation).” All data were analyzed with SPSS 25.0 (IBM, United States) and plotted using GraphPad Prism 8. Statistical significances were evaluated with the Student’s *t*-test (unpaired Student’s *t*-test, *P* < 0.05).

## Results

### Growth Characteristics of *Streptococcus bovis* S1 and Its *ccpA* Mutant at Different Glucose Levels

**FIGURE 1:**
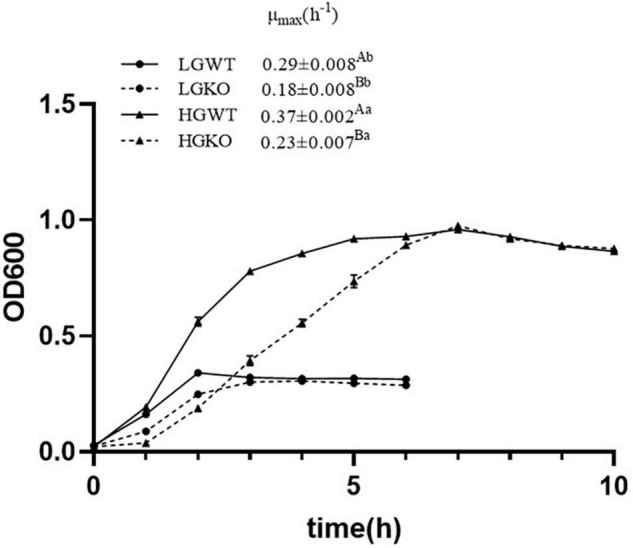
Growth curve of wild-type strain (WT) and *ccpA*-knockout strain (KO) of *S. bovis* S1 in glucose-excess or -limited conditions measured as optical density at 600 nm (OD_600_). Error bars indicate SD. The maximal growth rate (μ_max_) was estimated and shown in the figure. Values marked with different superscript uppercase letters (AB) indicate those are statistically significant differences (*P* < 0.05) between wild-type strain (WT) and *ccpA*-knockout strain (KO); values marked with different superscript lowercase letters (ab) indicate those are statistically significant differences (*P* < 0.05) between strains grown in glucose-excess or -limited conditions. LGWT, the wild-type strain grown in the media with 5 mM glucose; LGKO, the *ccpA*-knockout strain grown in the media with 5 mM glucose; HGWT, the wild-type strain grown in the media with 50 mM glucose; HGKO, the *ccpA*-knockout strain grown in the media with 50 mM glucose.

The growth curves for *S. bovis* S1 wild strain and the *ccpA* deletion strain under glucose-excess or -limited conditions are shown in Figure 1. In the stationary phase, as expected, the optical density (OD_600_) values of both strains in glucose-excess were significantly higher than those in glucose-limited conditions. Moreover, the growth yield of the two strains in same glucose levels exhibited no differences in the stationary phase. However, the inactivation of *ccpA* significantly reduced the maximal growth rate (μ_max_) of bacteria in the exponential phase (*P* < 0.05) in both glucose-excess (0.37 ± 0.002 h^–1^ versus 0.23 ± 0.007 h^–1^, respectively) and glucose-limited conditions (0.29 ± 0.008 h^–1^ versus 0.18 ± 0.008 h^–1^, respectively); μ_max_ of both the strains grown in glucose-excess were significantly higher than those in glucose-limited conditions (*P* < 0.05). These data suggested that the growth of *S. bovis* S1 was dependent on both CcpA and glucose availability.

### Fermentation Profiles of *Streptococcus bovis* S1 and Its *ccpA* Mutant at Different Glucose Levels

**FIGURE 2:**
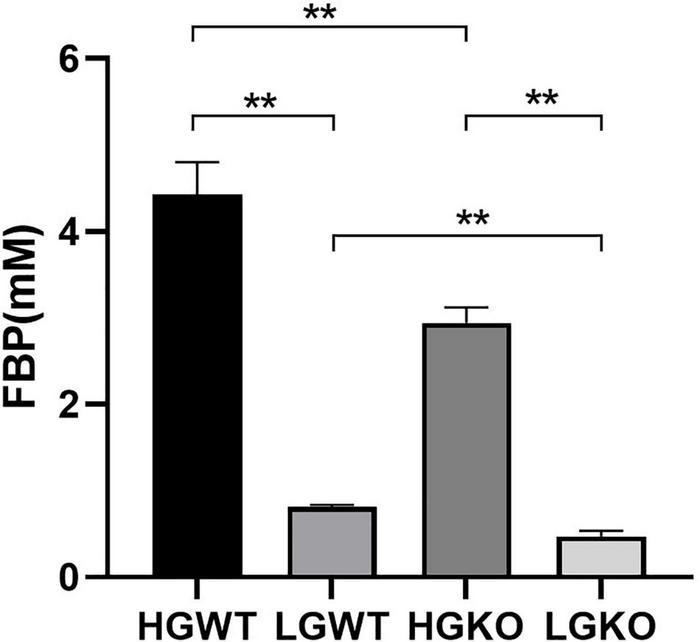
Intracellular concentration of FBP in wild-type strain (WT) and *ccpA*-knockout strain (KO) of *S. bovis* S1 in glucose-excess or -limited conditions. HGWT, the wild-type strain grown in the media with 50 mM glucose; HGKO, the *ccpA*-knockout strain grown in the media with 50 mM glucose; LGWT, the wild-type strain grown in the media with 5 mM glucose; LGKO, the *ccpA*-knockout strain grown in the media with 5 mM glucose. **Means *P* < 0.01.

Table 1 shows the organic acid production characteristics of *S. bovis* S1 wild-type and *ccpA* deletion strains in glucose-excess or -limited conditions. In the exponential growth phase, the lactate yield and percentage of *S. bovis* S1 wild-type strain were significantly higher than those of *ccpA* deletion strain in glucose-excess condition (*P* < 0.05); corresponding values for formate and acetate of wild-type strain were lower than those of the mutant strain (*P* < 0.05). Similar results were observed for organic acid production in the low-glucose conditions. Indeed, the yields of organic acids for both strains were greater in the high-glucose condition, however, the percentages of formate and acetate were much higher in glucose-limited culture. The observations in the stationary phase were similar to those in the exponential growth phase, except for the difference in acetate yield and percentage between both strains grown in low-glucose condition, which were statistically non-significant. As shown in Figure 2, the FBP concentration in *S. bovis* S1 wild-type strain was significantly higher than that of *ccpA* deletion strain in both the glucose-excess and glucose-limited conditions (*P* < 0.05); the concentrations of FBP for both strains were significantly higher in high-glucose conditions (*P* < 0.05).

**TABLE 1:** The organic acid production characteristics of *S. bovis* S1 wild-type strain and *ccpA*-knockout strain at the exponential and stationary phases in glucose-excess or -limited conditions.^1^

Conditions		Wild-type strain			*ccpA*-knockout strain		
		Lactate	Formate	Acetate	Lactate	Formate	Acetate
**The exponential phase^2^**							
HG	OA yields (mM)^3^	38.04 ± 0.529^Aa^	4.54 ± 0.248^Ba^	2.27 ± 0.067^Ba^	33.24 ± 0.546^Ba^	6.91 ± 0.295^Aa^	3.93 ± 0.335^Aa^
	OA percentages (%)^4^	84.83 ± 0.287^Aa^	10.12 ± 0.382^Bb^	5.06 ± 0.186^Bb^	75.41 ± 0.862^Ba^	15.67 ± 0.456^Ab^	8.92 ± 0.727^Ab^
LG	OA yields (mM)^3^	13.37 ± 0.118^Ab^	2.12 ± 0.045^Bb^	1.91 ± 0.067^Bb^	10.14 ± 0.059^Bb^	2.79 ± 0.181^Ab^	2.25 ± 0.010^Ab^
	OA percentages (%)^4^	76.82 ± 0.333^Ab^	12.18 ± 0.102^Ba^	10.99 ± 0.251^Ba^	66.77 ± 0.715^Bb^	18.38 ± 0.892^Aa^	14.85 ± 0.177^Aa^
**The stationary phase^2^**							
HG	OA yields (mM)^3^	84.06 ± 0.711^Aa^	9.05 ± 0.363^Ba^	4.15 ± 0.391^Ba^	66.77 ± 1.606^Ba^	15.97 ± 0.537^Aa^	10.91 ± 0.362^Aa^
	OA percentages (%)^4^	86.43 ± 0.306^Aa^	9.30 ± 0.316^Bb^	4.27 ± 0.427^Bb^	71.30 ± 0.761^Ba^	17.05 ± 0.445^Aa^	11.65 ± 0.317^Aa^
LG	OA yields (mM)^3^	13.79 ± 0.201^Ab^	2.18 ± 0.067^Bb^	2.11 ± 0.075^Ab^	12.46 ± 0.112^Bb^	3.07 ± 0.121^Ab^	2.25 ± 0.154^Ab^
	OA percentages (%)^4^	76.29 ± 0.359^Ab^	12.05 ± 0.230^Ba^	11.66 ± 0.119^Aa^	70.08 ± 0.911^Ba^	17.27 ± 0.459^Aa^	12.66 ± 0.792^Aa^

*^1^Data are presented as “mean ± SD (standard deviation).” Values marked with different superscript uppercase letters (AB) indicate those are statistically significant differences (P < 0.05) between wild-type strain (WT) and ccpA-knockout strain (KO); values marked with different superscript lowercase letters (ab) indicate those are statistically significant differences (P < 0.05) between strains grown in glucose-excess or -limited conditions.*

*^2^At the exponential phase, the values of OD_600_ is 0.6 and 0.2 in glucose-excess and -limited conditions, respectively; at the stationary phase, the values of OD_600_ is 0.9 and 0.3 in glucose-excess and -limited conditions, respectively.*

*^3^The concentrations of organic acids (mM), including lactate, formate, and acetate.*

*^4^Organic acids (mM) as a molar percentage of the total acid products (mM).*

### Transcriptomic Analysis of *Streptococcus bovis* S1 and Its *ccpA* Mutant Strains at Different Glucose Levels

To further investigate the global transcriptional regulation of CcpA in *S. bovis* S1 at different glucose concentrations, the transcriptome of *S. bovis* S1 wild-type and *ccpA* mutant were sequenced in glucose-excess or -limited conditions. RNA-seq was performed in the exponential growth phase of the bacterium. The statistical data for the transcriptome analysis is presented in Supplementary Table 1. A total of 364 million raw reads were generated from all samples. After the removal of low-quality reads, about 334 million clean reads with an average read length of 150 bp were obtained and used for subsequent analysis. Of these, 316 million were mapped onto the annotated *S. equinus* S1 genome with an average mapping ratio of at least 94.9%; the sequence reads matched with all the 1802 coding genes in the *S. equinus* S1 genome, which indicated that the sequencing depth was sufficient to cover all the transcripts in the cells (Supplementary Figure 2).

**FIGURE 3:**
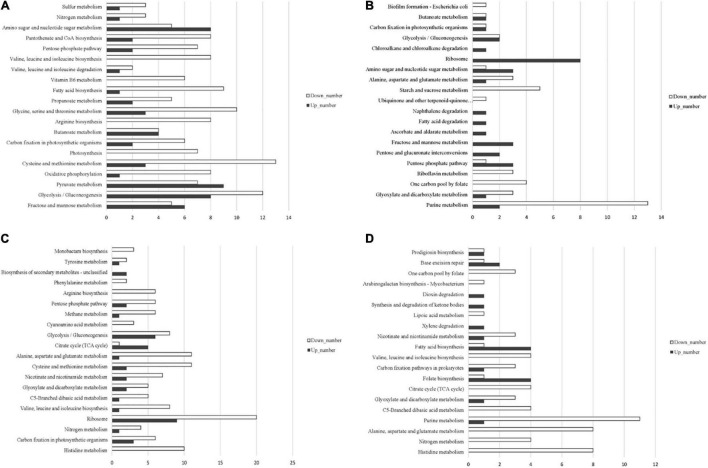
Distribution of upregulated and downregulated genes in the four pair-wise comparisons based on KEGG pathway categories. **(A)** The comparison between wild-type and its *ccpA* mutant grown in glucose-excess condition; **(B)** the comparison between wild-type and its *ccpA* mutant grown in glucose-limited condition; **(C)** the comparison between wild-type strain grown in glucose-excess and -limited conditions; **(D)** the comparison between *ccpA* mutant grown in glucose-excess and -limited conditions.

Based on the annotation of the *S. equinus* S1 genome, the read counts for each gene were calculated using HTSeq 0.6.1p2. The gene expression levels were normalized for different samples using FPKM. The gene expression profile of all samples was evaluated using principal component analysis (PCA). As expected, the three biological replicates cluster together closely for each treatment condition (Supplementary Figure 3). Differentially expressed genes (DEGs) were identified using the DESeq software with an absolute value of log_2_FoldChange > 1 and the false discovery rate (FDR) <0.05. DEGs between *S. bovis* S1 wild-type and its *ccpA* mutant in glucose-excess or -limited conditions were identified by four pair-wise comparisons of the overall transcriptome profiles as follows: between wild-type strains grown in glucose-excess and -limited conditions; between *ccpA* mutants grown in glucose-excess and -limited conditions; between wild-type and its *ccpA* mutant in glucose-excess conditions; between wild-type and its *ccpA* mutant in glucose-limited conditions (Supplementary Figure 4). Global gene expression patterns were visualized by volcano plots (Supplementary Figure 5). To better understand the molecular mechanisms of CcpA-regulated phenotypic differences of *S. bovis* S1 at different glucose concentrations, an enrichment analysis for the differential expressed genes was performed using the Kyoto Encyclopedia of Genes and Genomes (KEGG) (Figure 3 and Supplementary Table 3).

To validate the RNA-seq results, we randomly selected 10 genes and examined their transcript levels by qPCR (Supplementary Table 2). Although the magnitude of the genetic variation was different between the two analyses, the qPCR results showed similar trends in upregulations or downregulations as in the transcriptomic analysis. This confirmed the reliability of the transcriptome data.

### Comparison Between *Streptococcus bovis* S1 Wild-Type and *ccpA* Mutant Strains Grown in Glucose-Excess Conditions

**FIGURE 4:**
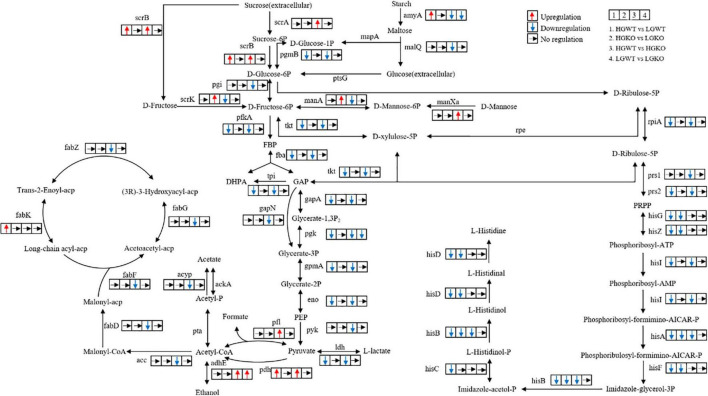
Overview of the key genes and related pathways changed in the transcriptomic analysis. 1, the comparison between wild-type strain grown in glucose-excess and -limited conditions; 2, the comparison between *ccpA* mutant grown in glucose-excess and -limited conditions. 3, the comparison between wild-type and its *ccpA* mutant grown in glucose-excess condition; 4, the comparison between wild-type and its *ccpA* mutant grown in glucose-limited condition. Gene annotation: scrA, sucrose PTS system EIIBCA or EIIBC component; scrB, beta-fructofuranosidase; scrK, fructokinase; amyA, alpha-amylase; malQ, 4-alpha-glucanotransferase; mapA, maltose phosphorylase; pgmB, beta-phosphoglucomutase; ptsG, glucose PTS system EIICB or EIICBA component; pgi, glucose-6-phosphate isomerase; manXa, mannose PTS system EIIA component; manA, mannose-6-phosphate isomerase; pfkA, 6-phosphofructokinase; fba, fructose-bisphosphate aldolase; tpi, triosephosphate isomerase; gapA, glyceraldehyde 3-phosphate dehydrogenase; gapN, glyceraldehyde-3-phosphate dehydrogenase (NADP+); pgk, phosphoglycerate kinase; gpmA, 2,3-bisphosphoglycerate-dependent phosphoglycerate mutase; eno, enolase; pyk, pyruvate kinase; ldh, L-lactate dehydrogenase; pfl, formate C-acetyltransferase; pdh, pyruvate dehydrogenase complex (pyruvate dehydrogenase E1 component, dihydrolipoamide dehydrogenase, pyruvate dehydrogenase E2 component); adhE, acetaldehyde dehydrogenase/alcohol dehydrogenase; pta, phosphate acetyltransferase; acyP, acylphosphatase; ackA, acetate kinase; acc, acetyl-CoA carboxylase; fabD, [acyl-carrier-protein] S-malonyltransferase; fabF, 3-oxoacyl-[acyl-carrier-protein] synthase II; fabG, 3-oxoacyl-[acyl-carrier protein] reductase; fabZ, 3-hydroxyacyl-[acyl-carrier protein] dehydratase; fabK, enoyl-[acyl-carrier protein] reductase II; tkt, transketolase; rpe, ribulose-phosphate 3-epimerase; rpiA, ribose 5-phosphate isomerase A; prs, ribose-phosphate pyrophosphokinase; hisG, ATP phosphoribosyltransferase; hisZ, ATP phosphoribosyltransferase regulatory subunit; hisI, phosphoribosyl-AMP cyclohydrolase; hisA, phosphoribosylformimino-5-aminoimidazole carboxamide ribotide isomerase; hisF, imidazole glycerol-phosphate synthase subunit; hisB, imidazoleglycerol-phosphate dehydratase; hisC, histidinol-phosphate aminotransferase; hisD, histidinol dehydrogenase.

A total of 822 differential genes were expressed in the wild-type and *ccpA* mutant strains grown in glucose-excess conditions. Among them, 401 genes were downregulated while 421 were upregulated. This comparison showed the largest number of genetic changes among the four comparisons studied. Sixty-one percent of these DEGs (502 genes) corresponded to 98 specific expression pathways, including metabolism pathways for carbohydrates, energy, and amino acids. Significantly altered carbohydrate metabolism pathways in KEGG following *ccpA* knockout were fructose and mannose metabolism, glycolysis/gluconeogenesis, pyruvate metabolism, and butanoate metabolism. In the fructose and mannose metabolism pathway (ko00051), four genes involved in fructose and mannose transport and gene encoding fructose-1-phosphate kinase were upregulated, while the genes encoding five enzymes involved in the metabolism of fructose and mannose were downregulated. These findings showed that CcpA regulated the transport and metabolism of fructose and mannose (Figure 4). The expression levels of 20 genes in the glycolysis/gluconeogenesis pathway (ko00010) were altered in absence of *ccpA* (Figure 4). In comparison with the wild-type strain, genes encoding the pyruvate dehydrogenase (PDH) complex, galactose mutarotase, phosphoenolpyruvate carboxykinase, and alcohol-acetaldehyde dehydrogenase were significantly upregulated in the *ccpA* mutant. The genes encoding L-LDH, phosphopyruvate hydratase, phosphoglycerate kinase, 6-phosphofructokinase, pyruvate kinase, two glyceraldehyde-3-phosphate dehydrogenases, 6-phospho-beta-glucosidases, triose-phosphate isomerase, phosphoglycerate mutase, and fructose-1,6-bisphosphate aldolase were significantly downregulated. We also identified DEGs involved in the pyruvate metabolism pathway (ko00620) (Figure 4). Among these DEGs, all the upregulated genes were also involved in the glycolysis/gluconeogenesis pathway except for those encoding for NAD-dependent malic enzyme, D-3-phosphoglycerate dehydrogenase, and formate acetyltransferase; genes encoding acetyl-CoA carboxylase biotin and acyl phosphatase were downregulated in the *ccpA* mutant. In addition, the inactivation of *ccpA* also led to the downregulation of the gene encoding α-amylase. Taken together, these results showed that deletion of *ccpA* affected the pathways in glycolysis and pyruvate metabolism of *S. bovis* S1. The downregulation of *ldh* and upregulation of *pfl* led to higher formate and lower lactate levels, which suggested that the absence of *ccpA* could switch the fermentation pattern from homolactic to mixed fermentation.

### Comparison Between *Streptococcus bovis* S1 Wild-Type and *ccpA* Mutant Strains in Glucose-Limited Conditions

We compared the transcriptomic profiles of wild-type and *ccpA* mutant strains in glucose-limited conditions. In this comparison, only 210 genes were significantly differentially expressed; 129 genes were upregulated while 81 genes were downregulated in the *ccpA* mutant. Compared with the transcriptomic comparison in glucose-excess condition, the DEGs reduced by approximately three quarters in glucose-limited condition. This indicated that the regulation of some genes by CcpA depended on glucose availability. Among these 210 DEGs, 118 could be grouped into different metabolic pathways in the KEGG database. In this comparison, only 4 significantly altered pathways were identified (*P* < 0.05), which included purine metabolism (ko00230), glyoxylate and dicarboxylate metabolism (ko00630), one carbon pool by folate (ko00670), and riboflavin metabolism (ko00740); only glyoxylate and dicarboxylate metabolism pathway was related to carbohydrate metabolism. Interestingly, there were almost no DEGs associated with pyruvate metabolism. Despite this, there were a significant number of DEGs involved in carbohydrate metabolism. The genes encoding 1-phosphofructokinase, glycosyl hydrolase family 32, PTS fructose transporter subunit IIC, galactose mutarotase, and alcohol-acetaldehyde dehydrogenase were upregulated in the absence of *ccpA*, while the genes encoding PTS cellobiose transporter subunit IIB, two alpha-amylases, glucose-6-phosphate isomerase, starch phosphorylase, phosphoglycerate kinase, and glucose-6-phosphate isomerase were significantly downregulated. These DEGs were mainly associated with fructose and mannose metabolism (ko00051), starch and sucrose metabolism (ko00500), and glycolysis/gluconeogenesis (ko00010), which was consistent with the results of comparison between the wild-type strain and *ccpA* mutant grown in glucose-excess condition. Notably, the expression of *ldh* and *pfl* was not significantly different in this comparison, which implied that the inactivation of *ccpA* might have little effect on the fermentation products in glucose-limited conditions.

### Comparison of *Streptococcus bovis* S1 Wild-Type Strain Grown in Glucose-Excess and -Limited Conditions

To examine the effects of glucose concentration at the transcriptional level in the *S. bovis* S1, the transcriptomes of wild-type strains grown in glucose-excess and -limited conditions were compared. A total of 680 genes were differentially expressed (*P* < 0.05): 316 genes were upregulated while 364 were downregulated in the wild-type strain grown in glucose-limited conditions. To assess their biological functions, 418 of the 680 DEGs were clustered in 92 special pathways in KEGG; 12 significant pathways were primarily involved in the metabolism of amino acids, energy, carbohydrates, and cofactors and vitamins. Here, we focused on the KEGG pathways associated with carbohydrate metabolism. Compared to the wild-type strain grown at high-glucose concentrations, the DEGs involved in the citrate cycle including genes encoding phosphoenolpyruvate carboxykinase and the PDH complex were significantly upregulated in the low-glucose condition, while the gene encoding citrate synthase was downregulated. The glycolysis/gluconeogenesis pathway in the *S. bovis* S1 wild-type strain was dependent on glucose concentrations in the culture. In this pathway, the upregulated genes were consistent with those in the citrate cycle pathway except for the gene encoding galactose mutarotase. The genes encoding enzymes including L-LDH, type I glyceraldehyde-3-phosphate dehydrogenase, phosphoglycerate kinase, 6-phosphofructokinase, phosphopyruvate hydratase, triose-phosphate isomerase, phosphoglycerate mutase, and fructose-1,6-bisphosphate aldolase were downregulated in low-glucose conditions. Besides, the low-glucose condition also upregulated the expression of genes encoding alpha-amylase, pyruvate formate lyase-activating protein, as well as CcpA. All of these results indicated that, although to a lesser extent, glucose availability could affect the fermentation pattern by transcriptional regulation in *S. bovis* S1 wild-type strain. This was also confirmed by the fermentation profiles.

### Comparison of *Streptococcus bovis* S1 *ccpA* Mutants Grown in Glucose-Excess and -Limited Conditions

Finally, the transcriptome profiles of *ccpA* mutant strains grown in glucose-excess and -limited conditions were compared. Here, a total of 340 genes were differentially expressed; 144 genes were upregulated while 196 were downregulated; 210 genes were annotated to different KEGG pathways. Unlike in the wild-type strain, no gene in the citrate cycle pathway was significantly upregulated; the gene encoding phosphoenolpyruvate carboxykinase and a cluster of three genes that encoded aconitate hydratase, citrate synthase and NADP-dependent isocitrate dehydrogenase were significantly downregulated in this comparison. Besides, the number of DEGs in pyruvate metabolism, starch and sucrose metabolism, fructose and mannose metabolism, and glycolysis was considerably lesser than the comparison of wild-type strains grown in glucose-excess and -limited conditions. These findings suggested that CcpA mediated the differential expression of these genes. The gene encoding L-LDH was downregulated in this comparison, which was consistent with the results of the comparison in wild-type strain. This result indicated that in low-glucose conditions, the yield of lactate of *S. bovis* S1 could reduce even in the absence of *ccpA*.

## Discussion

In the ruminants, inhibition of the overgrowth of *S. bovis* in the rumen is critical for the prevention of rumen acidosis (Wang et al., 2015). In the present study, deletion of *ccpA* resulted in a decreased growth rate of *S. bovis* S1, which implied that the overgrowth of *S. bovis* could be inhibited by controlling CcpA synthesis, consequently relieving rumen acidosis. The results are consistent with the results obtained in other lactic acid-producing bacteria, such as *Lactobacillus bulgaricus* (Li et al., 2016) and *Lactobacillus casei* (Esteban et al., 2004), although the growth differences between *S. bovis* 12U1 wild strain and *ccpA*-disrupted mutants have not been previously observed (Asanuma et al., 2004a). Previous study reported that CcpA regulated the growth rate of bacteria depending on the extracellular glucose concentration in *Streptococcus intermedius* (Imaki et al., 2014). However, in the present study, we observed a reduced growth due to the inactivation of *ccpA* in both glucose-excess and glucose-limited conditions.

In addition, suppressing the overproduction of lactate by *S. bovis* is also important to prevent rumen acidosis. Bacteria can shift fermentation patterns to produce lactate instead of mixed acids depending on the growth condition (McLeod et al., 2017). In the present study, we observed an inconsistent fermentation pattern for organic acids in *S. bovis* S1 grown in glucose-excess and glucose-limited conditions; fermentation products, including lower acetate and formate and higher lactate percentages, were obtained in excess-glucose conditions, which was consistent with the results of Chen et al. (2016a). This finding suggested that increasing glucose concentrations could induce a shift in fermentation patterns from heterofermentation to homofermentation in *S. bovis* S1. This is also observed in other lactic acid bacteria (Thomas et al., 1979; Bitoun et al., 2012; McLeod et al., 2017). CcpA has been reported to mediate the transcription of the gene encoding LDH (*ldh*) (Luesink et al., 1998; van den Bogaard et al., 2000; Asanuma et al., 2004a), PFL (*pfl*) (Asanuma et al., 2004a; Reed et al., 2018), and acetate kinase (*ack*) (Wunsche et al., 2012; Kim and Burne, 2017; Reed et al., 2018), thereby altering the production of organic acids. Here, we showed that the deletion of *ccpA* led to an increase in the percentage of acetate and formate and a significant reduction in lactate percentage. Notably, there was over a 10% reduction of lactate production in the *ccpA*-mutant in both exponential and stationary phases in glucose-excess conditions; while in glucose-limited conditions, only a 7% decrease was observed. The results of fermentation end-products indicated that CcpA could be a key factor for changes in fermentation pattern of *S. bovis* S1, and this was dependent on the glucose concentration.

In the EMP pathway, glucose-6-phosphate is converted to FBP by glucose-6-phosphate isomerase and phosphofructokinase, and FBP is then split into GAP and DHAP by FBP aldolase (FBA) (Bond and James, 1998). The intracellular FBP concentration fluctuates depending on the rate of glucose influx (Asanuma and Hino, 2000, 2002a). When *S. bovis* is growing in the presence of excess glucose, the intracellular FBP concentration is high, and as the availability of glucose decreases, the intracellular FBP concentration also decreases (Bond and James, 1998). Correspondingly, we observed a decrease in FBP in *S. bovis* S1 growing in glucose-limited media as compared to glucose-rich media. LDH activity can be induced by FBP in *S. bovis* (Wolin, 1964), and FBP concentration is positively correlated with the levels of *ldh*-mRNA and inversely correlated with the *pfl*-mRNA levels (Asanuma et al., 2004b). In this study, the increased lactate and decreased formate production in glucose-rich media might be associated with the increased FBP concentration. In addition, the concentration of FBP was reduced in *ccpA* deletion strains, which might be attributed to the negative effect of *ccpA* inactivation on the glycolysis process.

Regulation of CcpA on the target genes is dependent on glucose level in *Clostridium difficile* (Antunes et al., 2012) and *Streptococcus mutans* (Kim et al., 2019). To better understand the transcriptional regulation of CcpA with glucose concentration in *S. bovis* S1, we performed a transcriptomic analysis of *S. bovis* S1 wild-type and *ccpA* mutant strains at different glucose levels. Among the four pair-wise comparisons, the DEGs were the highest between *S. bovis* S1 wild-type and *ccpA* mutant strains grown at high glucose concentrations and were the lowest between these two strains grown in low glucose concentrations. The findings showed that CcpA could indeed regulate the gene transcription of *S. bovis* S1 depending on the extracellular glucose concentration. This was consistent with our metabolite analyses. Also, these results implied that controlling the growth and metabolism of *S. bovis* by CcpA may have great potential for prevention of rumen acidosis in ruminants fed a high-concentrate diet. Moreover, the DEGs in *S. bovis* S1 wild-type grown in glucose-excess and glucose-limited conditions were twice as high as those of *ccpA* mutants. This indicated that the gene expression of *S. bovis* S1 was dependent on extracellular glucose concentration, and the observed effect was attenuated by the inactivation of *ccpA*.

Glucose is metabolized to pyruvate *via* glycolysis after uptake, then pyruvate is further converted to lactate, formate, acetate, and ethanol in *S. bovis* (Asanuma and Hino, 2000). Interestingly, the transcriptomic analyses in this study focused on glycolysis and pyruvate metabolism, especially in the comparison between *S. bovis* S1 wild-type strain grown in glucose-excess and glucose-limited conditions and the comparison between *S. bovis* S1 wild-type and *ccpA* mutant strains grown at high glucose concentrations. For *S. bovis* S1 wild-type strain, most PDH complex genes were significantly upregulated in low glucose conditions, and genes related to the glycolysis and lactate production pathways were downregulated. This was consistent with the result for metabolites, where low glucose concentrations altered the metabolism of *S. bovis* S1 wild-type strain to produce less lactate. In lactic acid bacteria, the metabolic shifts during growth are associated with multiple factors, among which the intracellular redox potential reflected by NADH/NAD^+^ ratio is a key sensor (Goel et al., 2012; van Hoek and Merks, 2012). It is reported that *ldh* appears to be regulated by the global regulators Rex and CcpA in *Enterococcus faecalis* (Opsata et al., 2010; Mehmeti et al., 2011), and both of which are sensitive to NADH/NAD^+^ levels. In this study, though we observed a reduced transcription level of *rex* and elevated *ccpA* in response to glucose limitation, the repression or activation of these two transcriptional regulators on target genes responding to glucose concentration need to be further investigated. In addition, herein, the *ldh* gene showed reduced expression, which probably was a consequence of low FBP concentration at low glucose concentration (Abbe et al., 1982). Interestingly, the differential expressions of those genes were eliminated after the *ccpA* gene deletion, which indicated that CcpA played an important regulatory role at different levels of glucose. Notably, despite glucose being the main carbon source for *S. bovis* S1 in the present study, the genes involved in starch degradation including starch phosphorylase and alpha-amylase were significantly upregulated in low glucose conditions. This phenomenon might be a potential response of the bacteria to insufficient energy resources.

Catabolite control protein A is a pleiotropic regulator involved in controlling in carbon metabolism in many bacteria species in response to changes in overall energy levels and amount of carbohydrate (Abranches et al., 2008; Willenborg et al., 2014; Hofmann et al., 2021). For example, >80% of the genes are controlled by CcpA in response to glucose in *Bacillus subtilis* (Moreno et al., 2001). The results of the present study showed that the genes involved in the pyruvate metabolism were upregulated for the production of formate, while downregulated for lactate production in absence of *ccpA*, which was consistent with the alteration in metabolite levels in *ccpA* mutants grown in both glucose-excess or glucose-limited conditions. These results implied that the fermentation patterns of *S. bovis* S1 could be regulated by CcpA *via* modifying the enzyme transcription, similar to the previous findings in other lactic acid bacteria (Lu et al., 2018; Chen et al., 2019). Transcription of acetate kinase is enhanced in *ccpA* mutants of *Lactobacillus plantarum* (Lu et al., 2018) and *S. mutans* (Kim and Burne, 2017). Interestingly, though the transcriptomic showed non-significant differences in acetate kinase levels between *S. bovis* S1 wild-type and *ccpA* mutant strains, the qPCR results showed an elevated expression of acetate kinase after *ccpA* inactivation at the high glucose concentrations, which validated the metabolic shifts. However, this elevation was attenuated when cells were grown at low glucose concentrations, which suggested that the acetate kinase regulation by CcpA depended on glucose availability. Furthermore, the knockout of *ccpA* also largely influenced fructose and mannose metabolism pathway of *S. bovis* S1 grown under glucose-excess condition; genes involved in the transportation of fructose and mannose were upregulated, and those related to the metabolism of fructose and mannose were downregulated. However, this effect was also attenuated when cells were grown in the low-glucose conditions. As found in *C. difficile*, the attenuated regulation of target genes by CcpA in the low-glucose conditions may point toward inactive CcpA regulatory systems of *S. bovis* S1 at low glucose concentrations (Antunes et al., 2011, 2012).

Apart from carbon metabolism, the glucose concentrations also influenced histidine metabolism and nitrogen metabolism in both *S. bovis* S1 wild-type and *ccpA* mutant strains. Almost all the genes in these pathways were downregulated at the high glucose concentrations, which suggested that the amino acid metabolism and nitrogen metabolism of *S. bovis* S1 were also altered in response to extracellular glucose concentration. Fatty acids are essential components of membranes in all organisms, and their biosynthesis and degradation are important for maintaining membrane lipid homeostasis in response to environmental changes (Fujita et al., 2007; Tojo et al., 2011). It has been reported that CcpA regulates the fatty acid metabolism in *B. subtilis* and *S. mutans* in response to environmental changes (Tojo et al., 2011; Faustoferri et al., 2015). In our present study, nine DEGs involved in fatty acid biosynthesis were downregulated in *ccpA* mutant as compared to the wild-type strains at high glucose concentrations, similar to the previous findings in *L. plantarum* (Lu et al., 2018). Interestingly, this phenomenon was abolished when cells were grown in glucose-limited conditions. These results indicated that the absence of *ccpA* attenuated the fatty acid synthesis of *S. bovis* S1 only when the energy was sufficient.

## Conclusion

In conclusion, high glucose concentration in the media led to the rapid growth of *S. bovis* S1 and a shift to produce more lactate. The inactivation of *ccpA* slowed down the proliferation of *S. bovis* S1 and shifted the fermentation pattern toward the production of less lactate and more formate and acetate. The whole-transcriptome analyses showed that the pathways for histidine metabolism, nitrogen metabolism, and carbohydrate metabolism were significantly altered in response to glucose concentration. CcpA was involved in the regulation of metabolic processes, including glycolysis, pyruvate metabolism, fructose- and mannose-metabolism, and fatty acid biosynthesis in *S. bovis* S1. The transcriptional regulation by CcpA was more potent for *S. bovis* S1 grown in glucose-excess conditions.

## Data Availability Statement

The datasets presented in this study can be found in online repositories. The names of the repository/repositories and accession number(s) can be found below: https://www.ncbi.nlm.nih.gov/Traces/study/?acc=PRJNA746134.

## Author Contributions

YJ designed and conducted the experiments, analyses, and wrote the manuscript. YF, HS, and YZ helped to collect the samples. HW directed in experiments design and wrote the manuscript. All authors contributed to the article and approved the submitted version.

## Conflict of Interest

The authors declare that the research was conducted in the absence of any commercial or financial relationships that could be construed as a potential conflict of interest.

## Publisher’s Note

All claims expressed in this article are solely those of the authors and do not necessarily represent those of their affiliated organizations, or those of the publisher, the editors and the reviewers. Any product that may be evaluated in this article, or claim that may be made by its manufacturer, is not guaranteed or endorsed by the publisher.

## Footnotes

1
http://bowtie-bio.sourceforge.net/index.shtml
