# Supplementary material for: Transcriptome Analysis Reveals Catabolite Control Protein A Regulatory Mechanisms Underlying Glucose-Excess or -Limited Conditions in a Ruminal Bacterium, Streptococcus bovis
Source: Front Microbiol. 2021 Nov 18;12:767769. doi: 10.3389/fmicb.2021.767769 (PMC8637274; doi:10.3389/fmicb.2021.767769)

**Transcriptome analysis reveals CcpA regulatory mechanisms underlying glucose-excess or -limited conditions in a ruminal bacterium, *Streptococcus bovis***

Yaqian Jin, Yaotian Fan, Ying Zhang, Hongrong Wang, ^*^

Laboratory of Metabolic Manipulation of Herbivorous Animal Nutrition, College of Animal Science and Technology, Yangzhou University, Yangzhou 225009, P. R. China.

**^*^Correspondence:** Hongrong Wang, College of Animal Science and Technology, Yangzhou University, Yangzhou 225009, P. R. China. Tel.: +86-514-87979196; Fax: +86-514-8735044; ORCID: 0000-0002-4980-1082, E-mail: [hrwang@yzu.edu.cn](mailto:hrwang@yzu.edu.cn).

**Figure S1.** The result of PCR verification for wild type strain (WT) and *ccpA*-knockout strain (KO) of *S. bovis* S1.


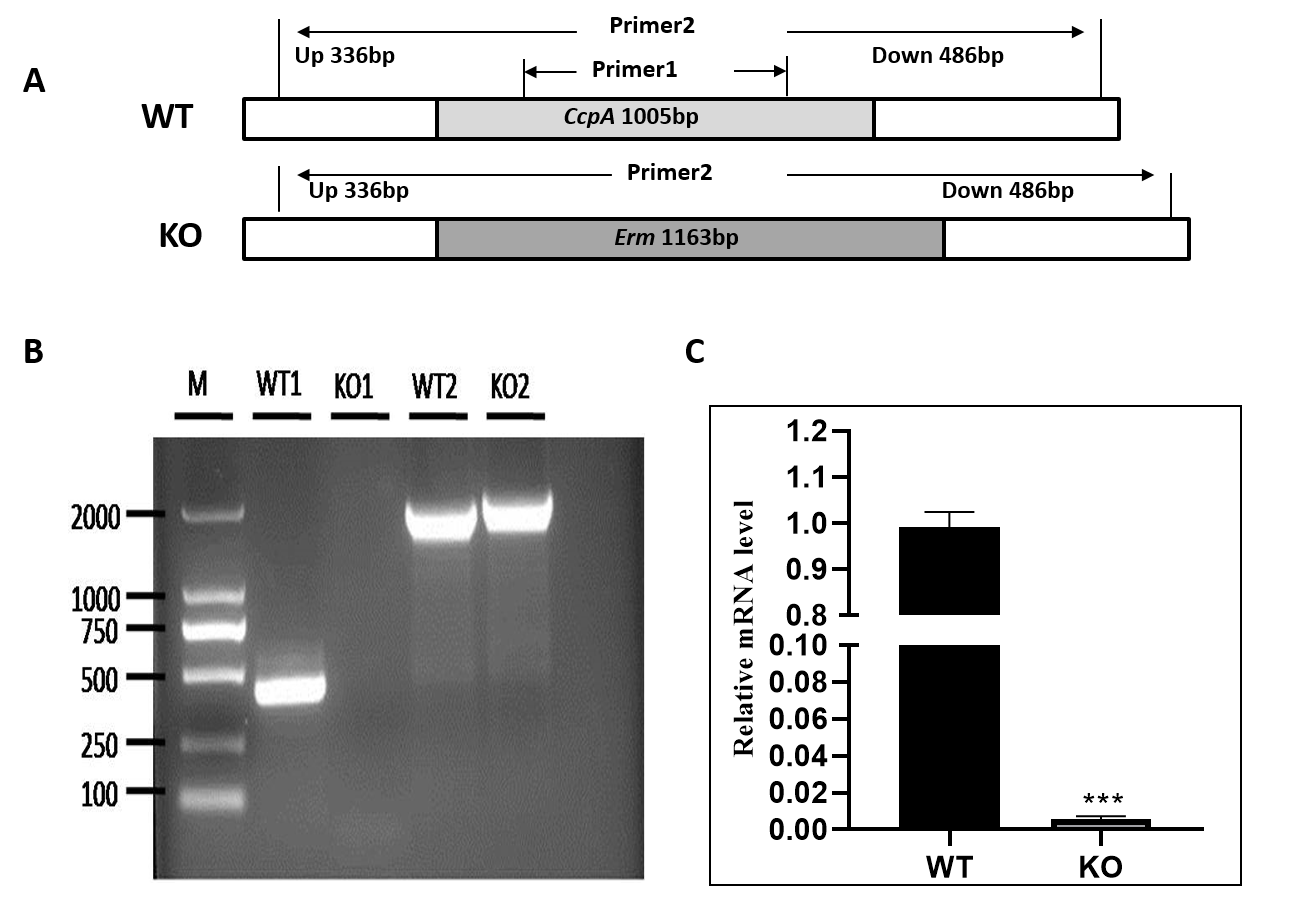


(A) Diagram of PCR verification for genomic structure of wild type strain (WT) and *ccpA*-knockout strain (KO). Primer 1 was designed based on the internal sequence of the *ccpA* gene, with a size of about 426bp; primer 2 was designed across the upstream and downstream sequences of the *ccpA* gene, with a size of about 1827bp for WT strain and 1985bp for KO strain. (B) The result of the PCR verification. WT1: a 426 bp PCR product of primer1 amplified in WT strain; KO1: no PCR product of primer1 amplified in KO strain; WT2: a 1827 bp PCR product of primer 2 amplified in WT strain; KO2: a 1985 bp PCR product of primer2 amplified in KO strain. (C) *ccpA* transcripts detected by qRT – PCR.

**Figure S2.** The sequencing saturation analysis of transcriptome data.


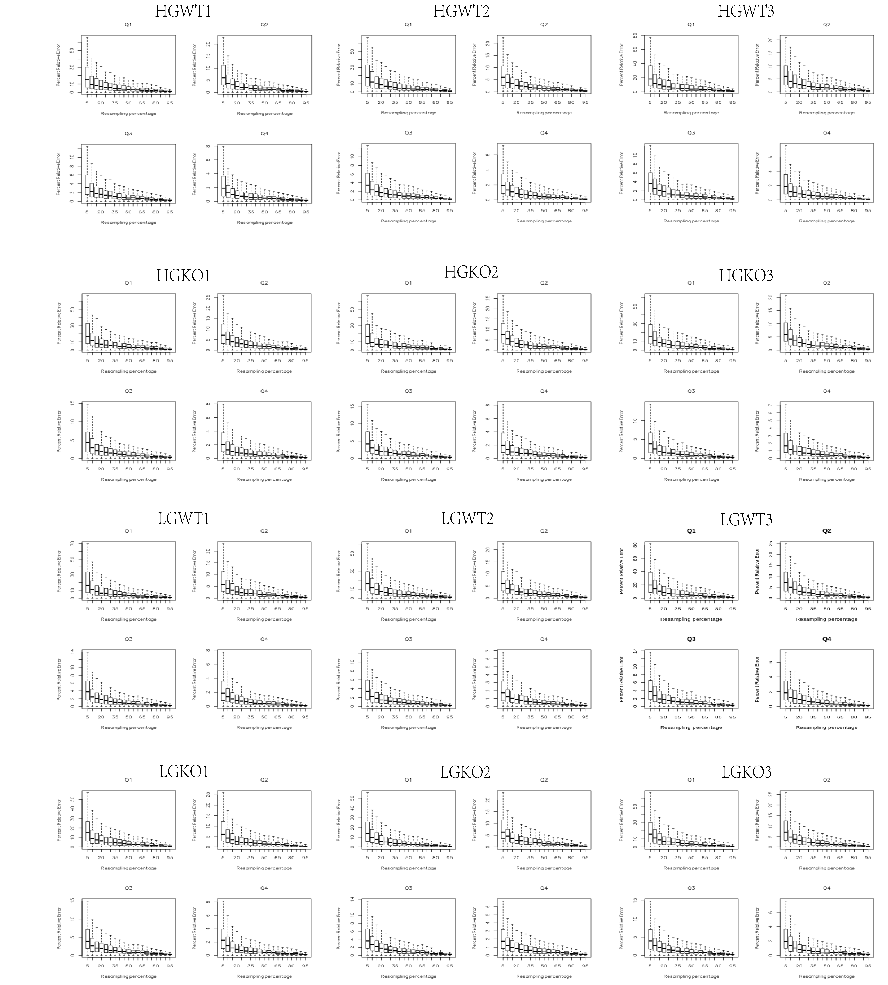


**Figure S3.** Principal component analysis on gene expression profile of four different groups.


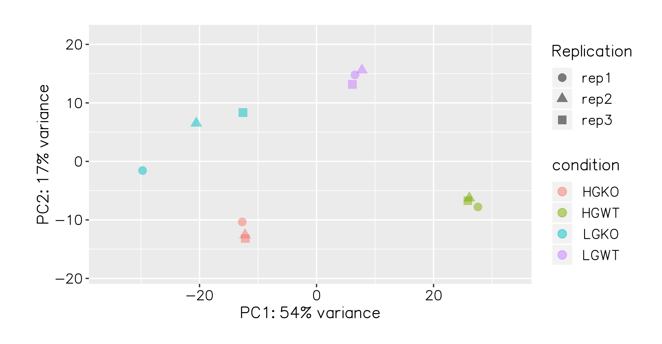


**Figure S4.** Venn diagrams of the number of genes differentially expressed in the four pair-wise comparisons.


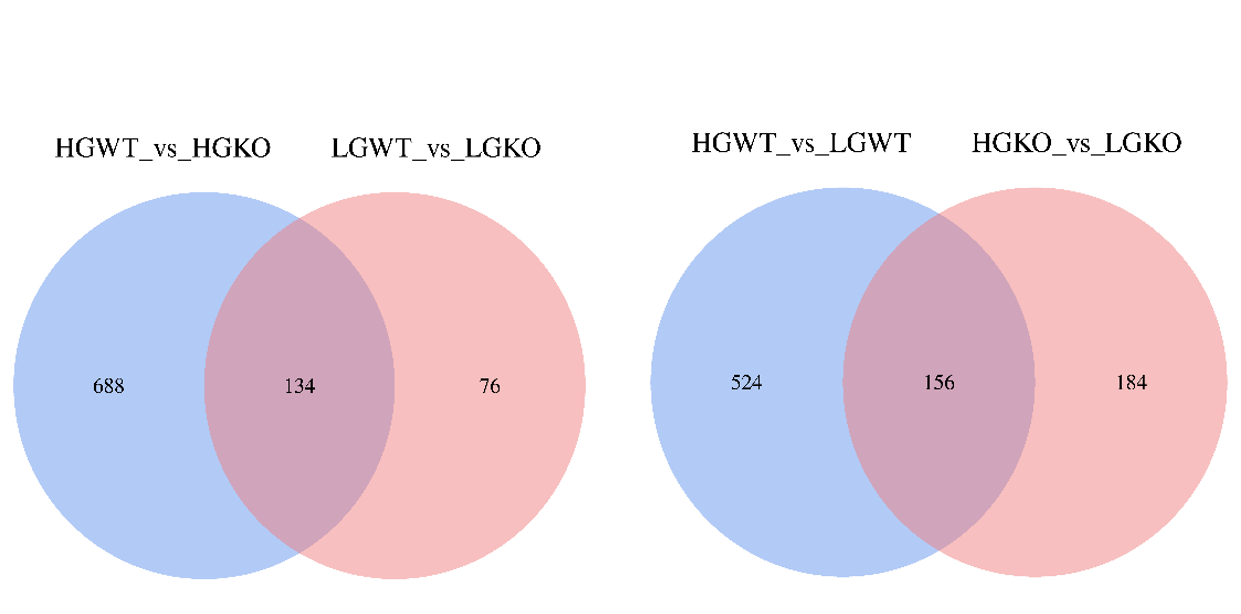


HGWT vs HGKO means the comparison between wild-type and its *ccpA* mutant grown in glucose-excess condition; LGWT vs LGKO means the comparison between wild-type and its *ccpA* mutant grown in glucose-limited condition; HGWT vs LGWT means the comparison between wild-type strain grown in glucose-excess and -limited conditions; HGKO vs LGKO means the comparison between *ccpA* mutant grown in glucose-excess and -limited conditions.

**Figure S5.** Volcano plots of gene expression.


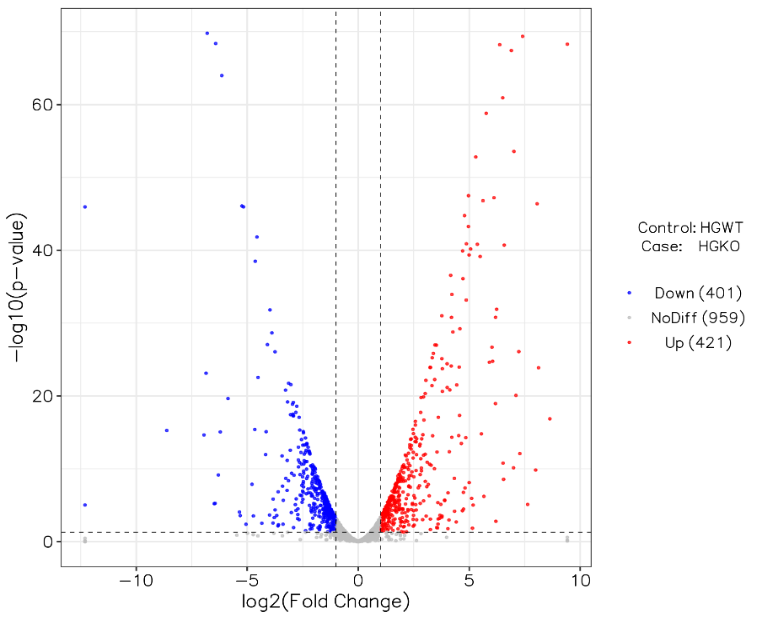

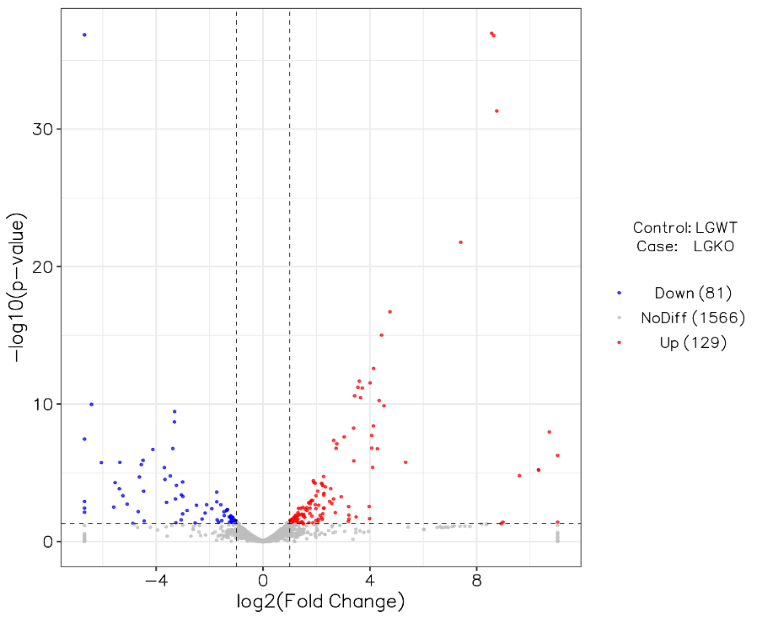

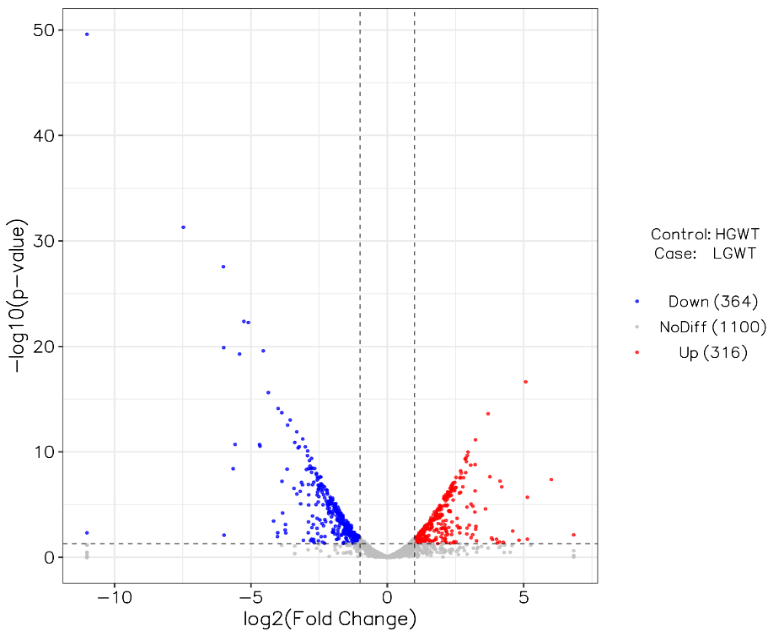

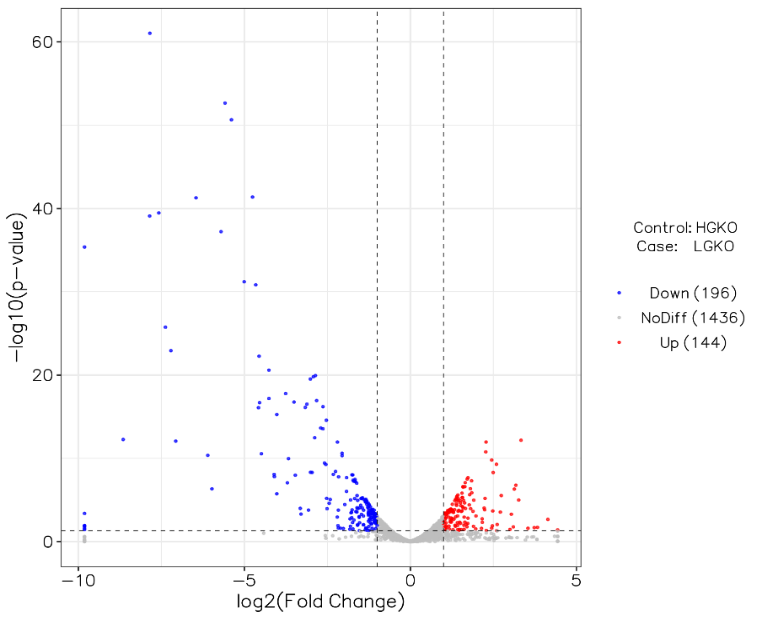

Supplement: Supplementary file 1 [file Data_Sheet_1.docx]
